# Supplementary material for: Phasor identifier: A cloud-based analysis of phasor-FLIM data on Python notebooks
Source: Biophys Rep (N Y). 2023 Nov 7;3(4):100135. doi: 10.1016/j.bpr.2023.100135 (PMC10694583; doi:10.1016/j.bpr.2023.100135)
Supplement: Document S1. Figures S1–S3 and Tables S1–S4 [file mmc1.pdf]

Biophysical Reports, Volume *3*

Supplemental information

Phasor identifier: A cloud-based analysis of phasor-FLIM data on Python notebooks

Mario Bernardi and Francesco Cardarelli

Supplementary Information

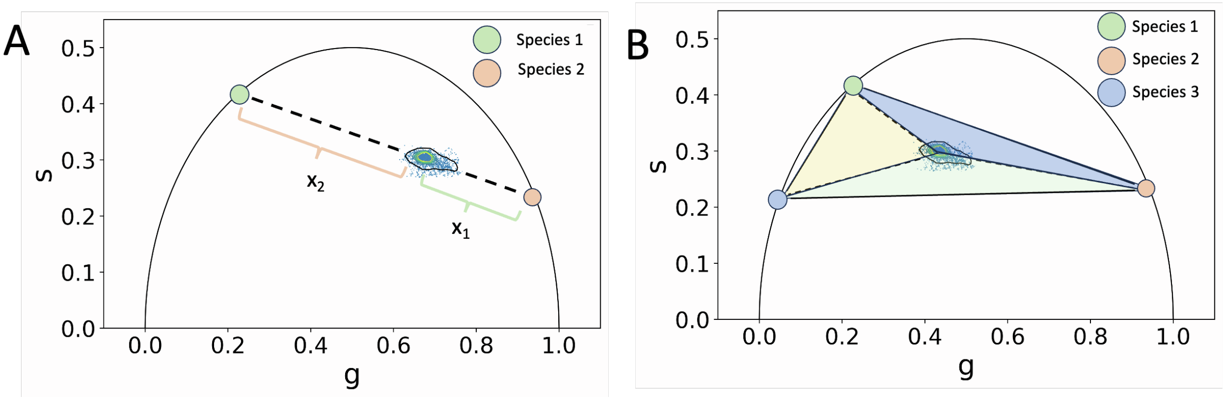

Figure S1. Schematic representation of the graphical approach for assessing intensity fractions of coexisting species knowing the phasor position of pure species a priori. A) In the case of two coexisting species, a linear approach enables straightforward calculation of the intensity fraction by considering the phasor's relative position along the characteristic segment segment. **B)** When dealing with three coexisting species, a linear approach facilitates the calculation of the intensity fraction based on the phasor's relative position within the characteristic triangle.

Sample	G	G std	S	S std	Lifetime [ns]	lifetime std [ns]	sampled points	Contour PCA variability ratio
**irinotecan pH3.6**	0.245019	0.007738	0.423314	0.006534	3.547728	0.010943	182338	0.581715
**irinotecan pH7.2**	0.238661	0.006967	0.421255	0.005676	3.595292	0.009852	170887	0.593733
**irinotecan pH8.4**	0.202396	0.006933	0.394718	0.005882	4.019535	0.009805	154348	0.579785
**SN-38 pH4.25**	0.282524	0.007482	0.403137	0.006235	3.517673	0.010582	244240	0.596726
**SN-38 pH7.2**	0.259005	0.008161	0.406107	0.006879	3.619584	0.011541	296999	0.589467

Table S1. Comprehensive overview of the phasor measurements of Figure 3. The data table shows the diverse impact of physical, chemical, and biological changes on irinotecan, revealing alterations in the FLIM signal.

pH	SN-38 lifetime [ns]	Irinotecan lifetime [ns]
**2.0**	1.98	3.65
**2.8**	2.97	3.61
**3.6**	3.44	3.54
**4.25**	3.50	3.55
**6.25**	3.52	3.56
**7.25**	3.59	3.57
**8.40**	3.89	4.01
**9.40**	4.00	3.99
**12.0**	4.21	4.02

Table S2. Comprehensive overview of the lifetime measurements of SN-38 and Irinotecan in PBS over the wide range of pH values under study, from 2 to 12. The data table shows the diverse impact of pH changes on the lifetime of SN-38 and Irinotecan, revealing alterations in fluorescence behaviour.

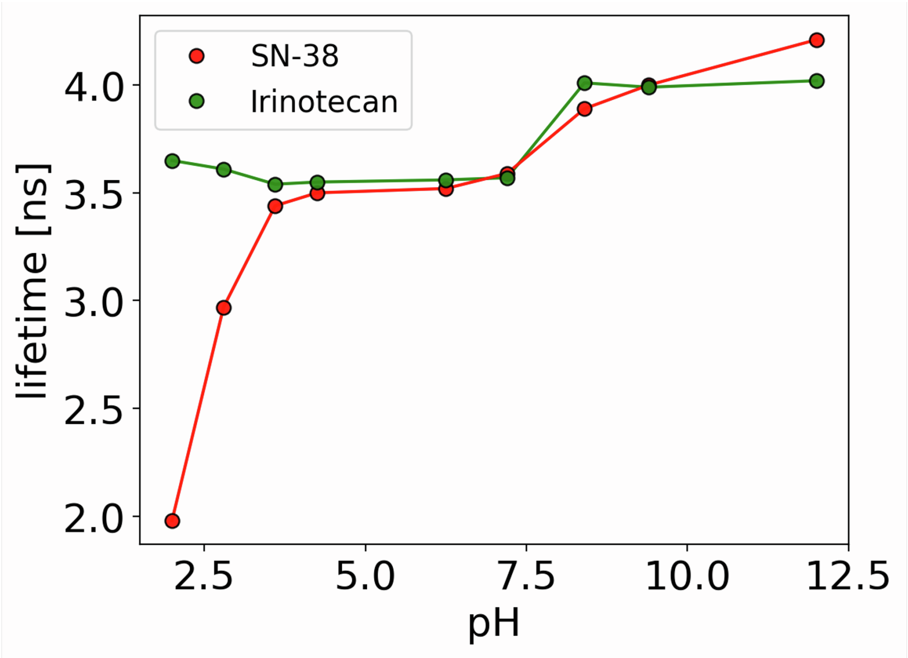

Figure S2. Comparison of the fluorescence lifetime of irinotecan (green) and SN-38 (red) at pH values between 2 and 12. Irinotecan exhibits minimal pH sensitivity compared to its metabolite.

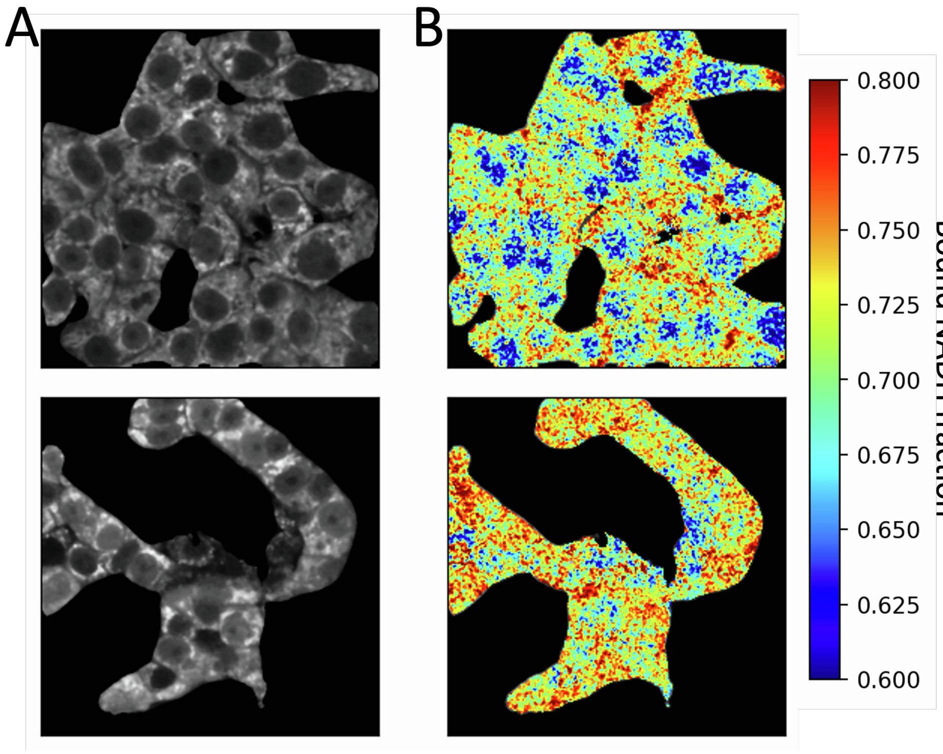

Figure S3: Distribution of Free and Bound NADH within INS1-E Cells Following Cytokine Treatment. A) Two-photon fluorescence intensity images of INS1-E cells under two conditions: control (above) and cytokine-treated (below). **B)** Mapping of the free and bound NADH levels in terms of the fraction of bound NADH in INS1-E cells under two conditions: control (above) and following cytokine treatment (below).

Distribution Parameter	Control	Cytokines
25th Percentile	2.4139	2.6355
50th Percentile	2.5383	2.7703
75th Percentile	2.669	2.9417
Central tendency average	2.5386	2.8161
Spread (Standard Deviation)	0.1921	0.2540
Skewness	-0.0434	0.9799
Mann Withney p-value	0.0	
Kolmogorov Smirnov p-value	1.3746e-86	

Table S3. Lifetime distribution data statistical comparison of INS1E cells (control) and Cytokines. Statistical analysis of the lifetime distributions of Figure 4.

Sample	ε [M^{-1} cm^{-1}]	QY %
Crystal doxorubicin	7510 ± 490	0.150 ± 0.004
Membrane-bound doxorubicin	10 340± 35	19.17 ± 0.4
Free-in-solution doxorubicin	10 340± 35	4.23 ± 0.09

Table S4. Quantum yield and molar extinction coefficient of doxorubicin in distinct physical states: crystal, membrane-bound, and free in solution. These values were sourced from reference (1) and were determined at the specific excitation wavelength of 488 nm.

References

1. Tentori, P., G. Signore, A. Camposeo, A. Carretta, G. Ferri, P. Pingue, S. Luin, D. Pozzi, E. Gratton, F. Beltram, G. Caracciolo, and F. Cardarelli. 2022. Fluorescence lifetime microscopy unveils the supramolecular organization of liposomal Doxorubicin. *Nanoscale*. 14:8901–8905.
