# Supplementary material for: Phasor identifier: A cloud-based analysis of phasor-FLIM data on Python notebooks
Source: Biophys Rep (N Y). 2023 Nov 7;3(4):100135. doi: 10.1016/j.bpr.2023.100135 (PMC10694583; doi:10.1016/j.bpr.2023.100135)
Supplement: Document S2. Article plus supporting material [file mmc2.pdf]

Biophysical Reports

ARTICLE

Phasor identifier: A cloud-based analysis of phasor-FLIM data on Python notebooks

Mario Bernardi[1,*] and Francesco Cardarelli[1,*]
[1]Laboratorio NEST, Scuola Normale Superiore, Pisa, Italy

ABSTRACT This paper introduces an innovative approach utilizing Google Colaboratory for the versatile analysis of phasor fluorescence lifetime imaging microscopy (FLIM) data collected from various samples (e.g., cuvette, cells, tissues) and in various input file formats. In fact, phasor-FLIM widespread adoption has been hampered by complex instrumentation and data analysis requirements. We mean to make advanced FLIM analysis more accessible to researchers through a cloud-based solution that 1) harnesses robust computational resources, 2) eliminates hardware limitations, and 3) supports both CPU and GPU processing. We envision a paradigm shift in FLIM data accessibility and potential, aligning with the evolving field of artificial intelligence-driven FLIM analysis. This approach simplifies FLIM data handling and opens doors for diverse applications, from studying cellular metabolism to investigating drug encapsulation, benefiting researchers across multiple domains. The comparative analysis of freely distributed FLIM tools highlights the unique advantages of this approach in terms of adaptability, scalability, and open-source nature.

WHY IT MATTERS?

Fluorescence lifetime imaging microscopy (FLIM) holds transformative potential in biology, enabling the study of diverse molecular processes. However, its widespread adoption is hindered by complex instrumentation and data analysis. This paper addresses these challenges by introducing an innovative approach using Google Colaboratory. This cloud-based solution provides robust computational resources, eliminates hardware limitations, and supports both CPU and GPU processing, making advanced FLIM analysis accessible to researchers. The result is a paradigm shift in FLIM data accessibility and potential, aligning with the evolving field of artificial intelligence-driven FLIM analysis. This approach simplifies FLIM data handling and opens doors for diverse applications, from cellular metabolism to drug encapsulation studies, benefiting researchers across multiple domains.

INTRODUCTION

Fluorescence lifetime imaging microscopy (FLIM) can be counted among the techniques with a transformative potential in the field of biological sciences, as in principle it enables the investigation and quantification of a multitude of molecular quantities and processes: exemplary applications encompass the measurement of intracellular parameters (e.g., metabolism (1–7), temperature (8–11), viscosity (12–14)), resolving the physical state of encapsulated fluorescent drugs (15–19), biomedical diagnostics (20–22), and neuroscience research (23–25). Yet, its widespread use and exploitation is still hampered by a number of limitations and bottlenecks. From a technological point of view, it should be noted that FLIM typically requires custom instrumentation, which is difficult to find embedded into commercial instruments. Another issue limiting the adoption of FLIM by a broad audience is the requirement for complex a-posteriori data analysis. Indeed, for decades, extracting quantitative information from FLIM data implied that lifetime decay curves were interpolated to a variety of mathematical models, in turn requiring extensive knowledge and expertise. In this regard, the idea to represent FLIM data in the Fourier space by the phasor plot represented a crucial achievement in the field (26–28). The phasor representation reduces the fluorescence lifetime decay to a vector in a polar plot (with information on the phase and modulation of the fluorescence lifetime). As such, it offers a graphical and intuitive vocabulary to guide data analysis

Submitted October 5, 2023, and accepted for publication November 3, 2023.

*Correspondence: mario.bernardi@sns.it or francesco.cardarelli@sns.it

Editor: Jorg Enderlein.

https://doi.org/10.1016/j.bpr.2023.100135

© 2023 The Authors.

This is an open access article under the CC BY license (http://creativecommons.org/licenses/by/4.0/).

TABLE 1 Comparative analysis of publicly available tools for FLIM (fluorescence lifetime imaging microscopy) data analysis

Software	Hardware	OS	Big data handling	Parallelization	Open source	Data formats
Phasor Identifier	not limited	not limited	suitable	suitable	completely	commercial* and SimFCS generated
SimFCS	limited to local CPU	Windows	not suitable	not suitable	no	commercial and proprietary
PAM	limited to local CPU	Windows MacOS	not suitable	not suitable	partially	proprietary only

The benchmarking process was conducted by aligning the tools with scientifically pertinent criteria, which were derived from the specific requirements of the FLIM field and contemporary data analysis standards. These criteria encompass computational power adaptability, operating system compatibility for analysis execution, flexibility concerning input file formats, data processing capabilities, and open-source nature. (*Note: currently, the tool reads .ifli raw data from VistaVision in addition to .ref and .R64 formats, with ongoing open-source development efforts to support various other formats.)

and interpretation in a model-free manner. In a microscopy image, for instance, pixels with similar decay curves will have similar coordinates in the phasor plot; also, pixels containing a combination of two (or more) distinct lifetime decays will be mapped according to the weighted linear combination of these contributions, in light of the so-called "phasor composition rules" originally introduced by Gregorio Weber in 1981 (29) and then refined by others (26,30,31). The inherently transformative nature of phasors is now also pushing at least two new research trends in the field: 1) optimization of the multicomponent-analysis strategies to move toward blind resolution of lifetime components in individual pixels (32–34) and 2) combination of lifetime to spectral phasor analysis to move toward a new paradigm of fast and robust spectral fluorescence lifetime imaging (35,36). In this rapidly evolving context, a lingering bottleneck still affects FLIM adoption by a broad audience, as recently pointed out by Prof. Leonel Malacrida (37); i.e., handling and processing the large amount of data generated by FLIM can be challenging, particularly for researchers without extensive expertise in data analysis. Although some commercial closed-source software packages (such as those provided by Becker & Hickl, PicoQuant, or Leica) and a few freely distributed ones (not always open source) such as SimFCS (31), FLUTE (38), and PAM (39) were developed to this aim, the handling and processing of large datasets in a quantitative, easy, fast, and interactive manner remains demanding in terms of resources.

In this context, we present an innovative approach that leverages the computational capabilities of Google Colaboratory (Colab) for state-of-the-art phasor-FLIM analysis (see Table 1). Google Colab provides access to robust computational resources, such as GPUs, substantial RAM, and ample disk space, thus obviating the necessity to make substantial investments in expensive hardware. This is particularly advantageous for those looking to seamlessly integrate artificial intelligence tools designed for FLIM (40–42) and work with big data.

Furthermore, our solution delivers a powerful tool to researchers, enabling them to operate within a cloud-based environment, effectively eliminating constraints related to computational power and operative system limitation. Our solution stands out for two key reasons: 1) it functions without any limitations associated with hardware, ensuring adaptability and scalability across a diverse range of computing resources. 2) It seamlessly supports both CPU and GPU processing, enabling parallelization, a feature that sets it apart from the software previously mentioned. These features align closely with the central objective of our research, which is to catalyze a paradigm shift in the accessibility and potential of FLIM data.

MATERIALS AND METHODS

Materials

Liposomal Irinotecan Onivyde was donated to Scuola Normale Superiore by the Medical Affair Department of Servier Italia. One 10-mL vial of sample contains 43 mg irinotecan anhydrous free base in the form of irinotecan sucrosofate salt in a pegylated liposomal formulation. The liposomal vesicle is composed of 1,2-distearoyl-sn-glycero-3- phosphocholine 6.81 mg/mL (1:1.6), cholesterol 2.22 mg/mL (1:0.5), and methoxy-terminated polyethylene glycol (MW 2000)-distearoylphosphatidyl ethanolamine (MPEG-2000-DSPE) 0.12 mg/mL (1:0.03). Each mL also contains 2-[4-(2-hydroxyethyl) piperazin-1-yl] ethanesulfonic acid (HEPES) as a buffer 4.05 mg/mL and sodium chloride as an isotonicity reagent 8.42 mg/mL. Irinotecan hydrochloride (powder), purchased from Sigma Aldrich (Milan, Italy), and Onivyde were both stored at 4°C in compliance with the datasheet. In this study, we evaluated the impact of pH variation on the characteristic lifetime of irinotecan and its metabolite SN-38, purchased from TCI Europe N.V. (Zwijndrecht, Belgium). The pH range studied was from 2.0 to 12.0, and the buffer used was PBS due to its compatibility with living cells and broad buffering capacity. To simplify the methodology, we opted to use PBS rather than more complex buffer mixtures, despite their higher buffering capacity. Nine PBS solutions were prepared with the desired pH, starting from stock solutions of irinotecan and SN-38 in DMSO. 1 mM solutions in PBS were then prepared for each pH point, and the final solutions were stirred to maintain the pH control. For additional insights into the case study on doxorubicin, readers are referred

to Tentori et al. (15). The Doxoves used in this study was purchased from FormuMax Scientific (Sunnyvale, CA, USA).

Cell culture

Insulinoma 1E (INS-1E) cells were a kind gift from Professor C. Wollheim from the University of Geneva. These cells were kept in a climate-controlled incubator set to 37°C and 5% CO_2, where they were grown in RPMI 1640 medium containing 11.1 mmol/L D-glucose, 10 mmol/L HEPES, 2 mmol/L L-glutamine, 100 U/mL penicillin-streptomycin, 1 mmol/L sodium-pyruvate, and 50 μmol/L β-mercaptoethanol. To conduct lifetime experiments, the cells were allowed to grow until they reached 70% confluence on sterilized microscopy-compatible dishes (IbiTreat μ-Dish 35-mm, Ibidi) for a period of 24–48 h. Then, the cells were exposed to either irinotecan or Onivyde diluted in complete medium. To serve as a control, the cells were simply refreshed with a fresh batch of complete medium.

FLIM measurements

A drop of approximately 20 μL of Onivyde was diluted 50x in 980 μL of saline per intravenous administration protocol. The solution was poured on the glass of a WillCo plate, without any further dilution. For what concerns the free drug, the 1 mM irinotecan stock solution in DMSO was diluted in different buffers before FLIM at a final concentration of ∼10 μM. Irinotecan precipitate and spin-coated liposomes were obtained on the glass of a WillCo plate and black glass-bottom 96-well plate, respectively, as described above. No aqueous solution was added before FLIM to avoid any possible drug re-suspension. FLIM measurements were performed by an Olympus FVMPE-RS microscope coupled with a two-photon Ti:sapphire laser with 80-MHz repetition rate (MaiTai HP, SpectraPhysics) and a FLIM box system for lifetime acquisition (ISS, Urbana Champaign) in digital frequency domain (DFD). Onivyde and irinotecan were excited at 760 nm, and the emission collected by using a 30X planApo silicon immersion objective (NA = 1.0) in the 380- to 570-nm range. Calibration of the ISS Flimbox system was performed by measuring the known monoexponential lifetime decay of fluorescein at pH = 11.0 (i.e., 4.0 ns upon excitation at 760 nm, collection range: 570–680 nm). To prepare the calibration sample, a stock of 100 mmol/L fluorescein solution in EtOH was prepared and diluted in NaOH at pH = 11.0 for each calibration measurement. For each measurement, a 512 × 512 pixels image of FLIM data was collected until 30 frames were acquired. In the context of phasor-FLIM metabolic investigations, living INS-1E cells were observed after 24 h of cytokine exposure in the standard maintenance conditions. These conditions involved RPMI 1640 medium supplemented with 11.1 mmol/L D-glucose, 10% heat-inactivated FBS, 10 mmol/L HEPES, 2 mmol/L L-glutamine, 100 U/mL penicillin-streptomycin, 1 mmol/L sodium-pyruvate, and 50 μmol/L β-mercaptoethanol, all maintained at 37°C. We then utilized two-photon excitation at 740 nm to capture images of the same cell clusters under both low and high glucose concentrations. Regarding the FLIM measurements related to doxorubicin, an approximately 50-μL droplet of Doxoves stock solution was carefully positioned on the glass surface of a WillCo plate. Notably, no further dilution was carried out. In this case, FLIM was performed using a Leica TCS SP5 confocal microscope (Leica Microsystems, Germany). A pulsed diode laser operating at a frequency of 40 MHz was employed for excitation at 470 nm. The emitted light was captured between 520 and 650 nm using a photomultiplier tube, which was linked to a time-correlated single-photon counting card from PicoQuant in Berlin, designated as PicoHarp 300. Raw data were internally processed into .bin format and then to .R64 format within SimFCS.

Phasor plot computations

Equations (1a) and (1b) describe the computation of the coordinates considering T, n, and ω, the period of the laser pulse, harmonic, and angular frequency, respectively.

$$g_{\{i,j\}}(\omega) = \int_0^T I(t)\cdot cos\,(n\omega t)dt \Big/ \int_0^T I(t)dt \quad (1a)$$

$$s_{\{i,j\}}(\omega) = \int_0^T I(t)\cdot sin\,(n\omega t)dt \Big/ \int_0^T I(t)dt \quad (1b)$$

In the frequency domain for each pixel, one can rely on modulation $m_{i,j}$ and phase shift $\varphi_{i,j}$ of the signal as reported in Eqs. (2a) and (2b):

$$g_{\{i,j\}} = m_{i,j}\cdot cos\,(\varphi_{i,j}) \quad (2a)$$

$$s_{\{i,j\}} = m_{i,j}\cdot sin\,(\varphi_{i,j}) \quad (2b)$$

The phasors lie within the semicircle, also known as "universal semicircle" (43–45), centered at (½,0) with radius ½ and positive x, where the zero lifetime is located at (1,0) and the infinite lifetime at (0,0). Specifically, in the instance of the point (0,0), the fluorescent species remains unexcited, resulting in an infinite lifetime. This is due to the absence of light emission, as the species does not experience excitation. On the other hand, at the coordinate (1,0), the fluorescence lifetime converges to zero, indicating a scenario in which the fluorescent species instantaneously reaches its ground state. In a concise explanation, it is noteworthy to underline that the fluorescence lifetime of a given species denotes the temporal duration during which the species remains in its excited state, whereas the amplitude represents the quantity of emitted light during the excitation phase. Indeed, by taking the Fourier transformation of a measured decay curve, the lifetime can be estimated relying solely on the position of the phasor inside the universal circle. The distribution of phasor points originating from FLIM measurements is found on the universal semicircle for monoexponential decays or within the universal semicircle for multiexponential decays (44,46). In fact, in the case of a monoexponential decay, the intensity can be expressed according to Eq. (3a), whereas multiexponential decay intensity can be expressed as a sum over multiple components. Specifically, a tri-exponential mode, Eq. (3b), often offers an effective representation for a wide array of fluorescence decay profiles, striking a balance between computational feasibility and accuracy. In Eq. (3b), subscripts f, b, and p indicate different physical states of the fluorescent compound (e.g., irinotecan in free, membrane-associated, and gelated/precipitated forms). Recent research endeavors have ventured into noncustomary scenarios where the computation of four components is explored (33). Notably, when considering more than three components, measurements at different harmonics become essential to accommodate the increased complexity of the analysis.

$$I_{mono}(t) = A_f e^{-t/\tau} \quad (3a)$$

$$I_{multi}(t) = A_f e^{-t/\tau_f} + A_b e^{-t/\tau_b} + A_p e^{-t/\tau_p} \quad (3b)$$

If two molecular species are coexisting in the same pixel, for instance, all the possible weighting combinations of the two molecular species give phasors distributed along a straight line joining the

characteristic phasors of the two pure species. In the case of three molecular species, all the possible combinations of the system form a triangle where the vertices correspond again to the characteristic phasors of the pure species.

Apparent lifetimes, associated with multiexponential component decay, can be determined using nonlinear least squares analysis of phase delay and modulation. Equations (4a) and (4b) describe the estimation of lifetimes in individual pixels with regard to both phase and modulation. Notably, monoexponential lifetimes produce identical values, whereas multiexponential lifetimes yield different results. This review does not cover the specific instrumentation or data analysis typically applied in time or frequency domain measurements.

$$\tau_{\varphi} = \frac{1}{\omega}\tan(\varphi) \tag{4a}$$

$$\tau_m = \frac{1}{\omega}\sqrt{\left(\frac{1}{m^2} - 1\right)} \tag{4b}$$

As shown in Fig. S1, given an experimental phasor that is the combination of two (or more) species and the phasors of the isolated pure components, intensity fractions can be graphically visualized and easily quantified.

RESULTS AND DISCUSSION

Phasor-FLIM data analysis: General workflow

In this study, experimental lifetime acquisitions underwent phasor analysis using custom Python 3.6 routines within the “Phasor Identifier” notebook in the Google Colab environment. The input parameters allow the specification of the file extension and analysis type, which can be configured as either cumulative, enabling the aggregation of multiple replica files into a single data set, or single file for the analysis of individual files. The file name format comprises the date, sample name, and replica number (e.g., 2023_experiment 1_1). As of today, allowed file formats are the following: “.R64,” “.ref,” and “.ifli.” These formats can be generated directly as raw data within commercial software for both DFD and frequency domain acquisitions or as processed data in the time domain. With the cumulative analysis setting, the code identifies samples by name and stitches images based on replica numbers. For each pixel in the FLIMbox-generated image, the fluorescence decays can be measured in the time domain (Fig. 1 *A*) and mapped onto a "phasor" plot (Fig. 1 *B*). The phasor plot has two coordinates, which are the real and imaginary parts of the Fourier transform of the fluorescence lifetime decay. This calculation is performed at the angular repetition frequency of the excitation laser. Consequently, pixels with similar decay curves exhibit similar coordinates on the phasor plot. Fig. 1 *B* demonstrates monoexponential decays, indicated by all points lying on the phasor plot. In the case of pixels containing a combination of two or more distinct lifetime decays, they are mapped based on the combination of these contributions. Interpreting the data in three dimensions (Fig. 1 *C*) is of utmost importance since there are inevitably regions in the phasor plot that exhibit varying degrees of population. Effective sampling of the phasor plot (47) goes beyond identifying an individual phasor position, contingent upon the specific characteristics of the sample. To facilitate an informed and appropriate selection of parameters, users are furnished with essential phasor characteristics. These include the number of sampled data points, indicating the analysis's significance, and the principal component analysis variability ratio of the identified region of interest (ROI), reflecting the phasor's directional qualities. These metrics can be harnessed to fine-tune the input parameters for the analysis. In Fig. 1 *C*, the characteristic luminescence signal is projected in 2D for visualization purposes and sampled with 100 bins in both dimensions. It is noteworthy that commonly available open-source codes generate contour plots using elliptical approximation of contour lines. However, this code analyzes the signal through frequency levels of a 3D distribution. The lowest level corresponds to the bottom frequency level attainable after intensity thresholding, and the top level identifies as the 95th percentile. Bottom and top level can be adjusted to facilitate frequency noise removal and improve the analysis quality.

The intensity threshold, as shown in Fig. 2 *A* on liposomal irinotecan (Onivyde) samples, can be set through three different approaches: custom threshold value, Otsu threshold, or multi-Otsu threshold. The default option is the multi-Otsu threshold, but setting a custom threshold would be straightforward as intensity values are normalized to 1 before data processing. Additionally, users have the option to import masks to restrict the analysis to a specific portion of the image, especially when focusing on a particular morphological feature; the code allows for masking cells via the cellpose (48) Python library also, leveraging deep learning-based segmentation method (in this case, it is advised to set the threshold to 0 to appreciate at the best the lifetime distribution within cells). Cellpose doesn't need extra training or adjusting settings, as it learned from over 70,000 cell images (49). It uses the common U-Net method to break down and rebuild images for accurate segmentation. Indeed, deep learning-driven segmentation methodologies have been readily deployed within the framework of FLIM analysis, yielding remarkable outcomes, as evidenced in pioneering studies pertaining to metabolic heterogeneity (50) and the impact of prolonged UVA-induced

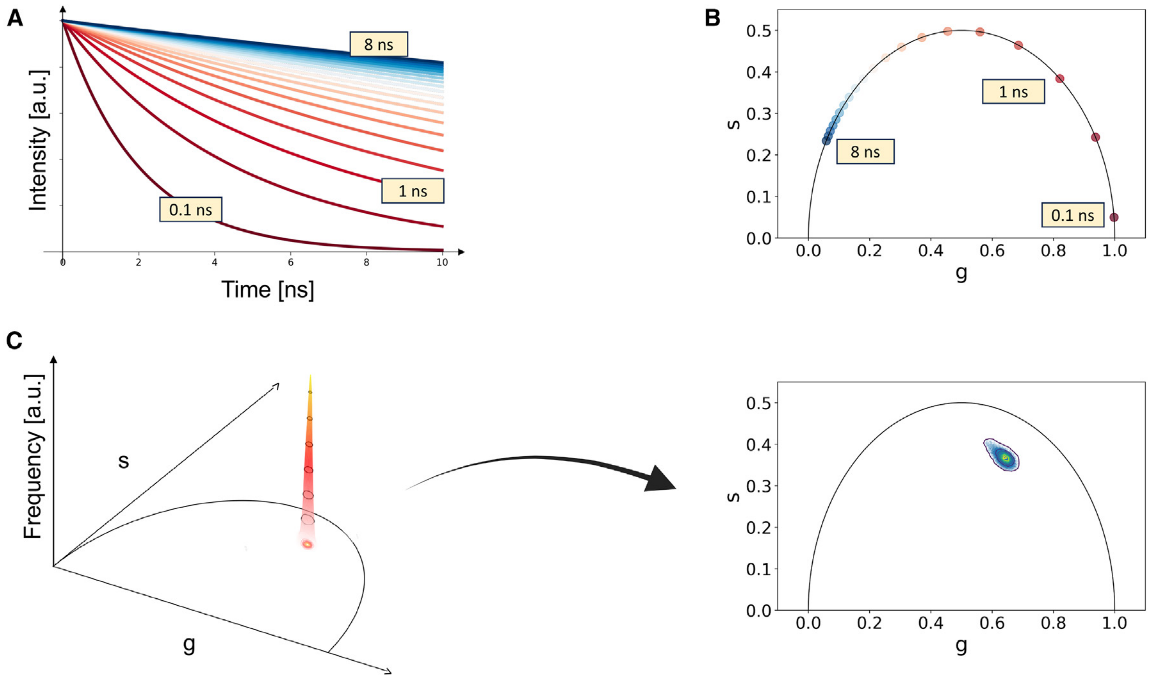

FIGURE 1 Phasor analysis of FLIM data. (*A*) A fluorescence decay is measured in the time domain in each pixel of the image. (*B*) The fluorescence lifetime decay Fourier transform coordinates are mapped onto a phasor plot, revealing distinct clusters for different decay patterns. (*C*) A 2D projection of the phasor plot helps visualize regions with varying population, highlighting the importance of interpreting the data in three dimensions for comprehensive analysis (the data shown are representative of a cellular phasor of encapsulated doxorubicin).

metabolic stress on reconstructed human skin (51). After setting the intensity threshold, the relevant pixels for the analysis are automatically detected. By saving the pixel numbers to be processed, access to the phase and magnitude matrices is restricted to only these relevant pixels, ensuring that the analysis focuses on the desired data points. These points undergo spatial domain filtering (Fig. 2 *B*), giving users the choice between a linear or nonlinear filter: namely Gaussian or median filter. The strength of a median filter refers to the window size used for filtering, where larger values yield stronger smoothing effects. Similarly, the strength of a Gaussian filter is determined by the standard deviation (sigma), with larger sigma values resulting in stronger smoothing. By default, the code sets a median filter strength to 3 x 3 as it is common practice in phasor-FLIM analysis (43). Furthermore, we have incorporated a "morphology and lifetime visualization" module to allow direct observation of how median filters and thresholding impact the sample. It's worth noting that applying an overly aggressive or an inappropriate median filter could inadvertently remove crucial spatial features (43) relevant to lifetime analysis, as well as employing excessive thresholding. The contour analysis function performs a comprehensive analysis of the phasor plot-generated FLIM data. It iterates through each frequency level detected in the 3D histogram (Fig. 2 *C*) and its corresponding contours, creating a Polygon object for each contour. It then checks if a contour belongs to a previously identified ROI or if it is a new one. A new ROI that satisfies the criteria for a valid contour is added to the perimeters list, which contains the contour data of the identified ROI, as shown in Fig. 2 *D*. Similarly, if the contour belongs to an existing ROI, the function updates the corresponding phasor if it is contained within any previously stored phasors; otherwise, it adds the contour as a new phasor as shown in Fig. 2 *E*. The valid contour criteria can be set manually. By default, new ROIs must contain at least 5000 data points, and new phasors must contain at least 500 data points. These default values are subject to adjustment based on the quality and nature of the sample being analyzed. As a result, the function generates two lists: phasors containing the contour data of identified subregions in the phasor plot and perimeters containing the contour data of identified ROIs. These lists provide a comprehensive representation of the ROIs and subregions in the phasor plot, facilitating further analysis and interpretation of the FLIM data. As reported in Fig. 2 *F*, the code maintains two distinct data frames: one, "df_dataset," storing each data point's data, and another, "df," documenting phasor properties. These data frames can be saved as CSV files and accessed beyond the Google Colab notebook for further analysis and visualization.

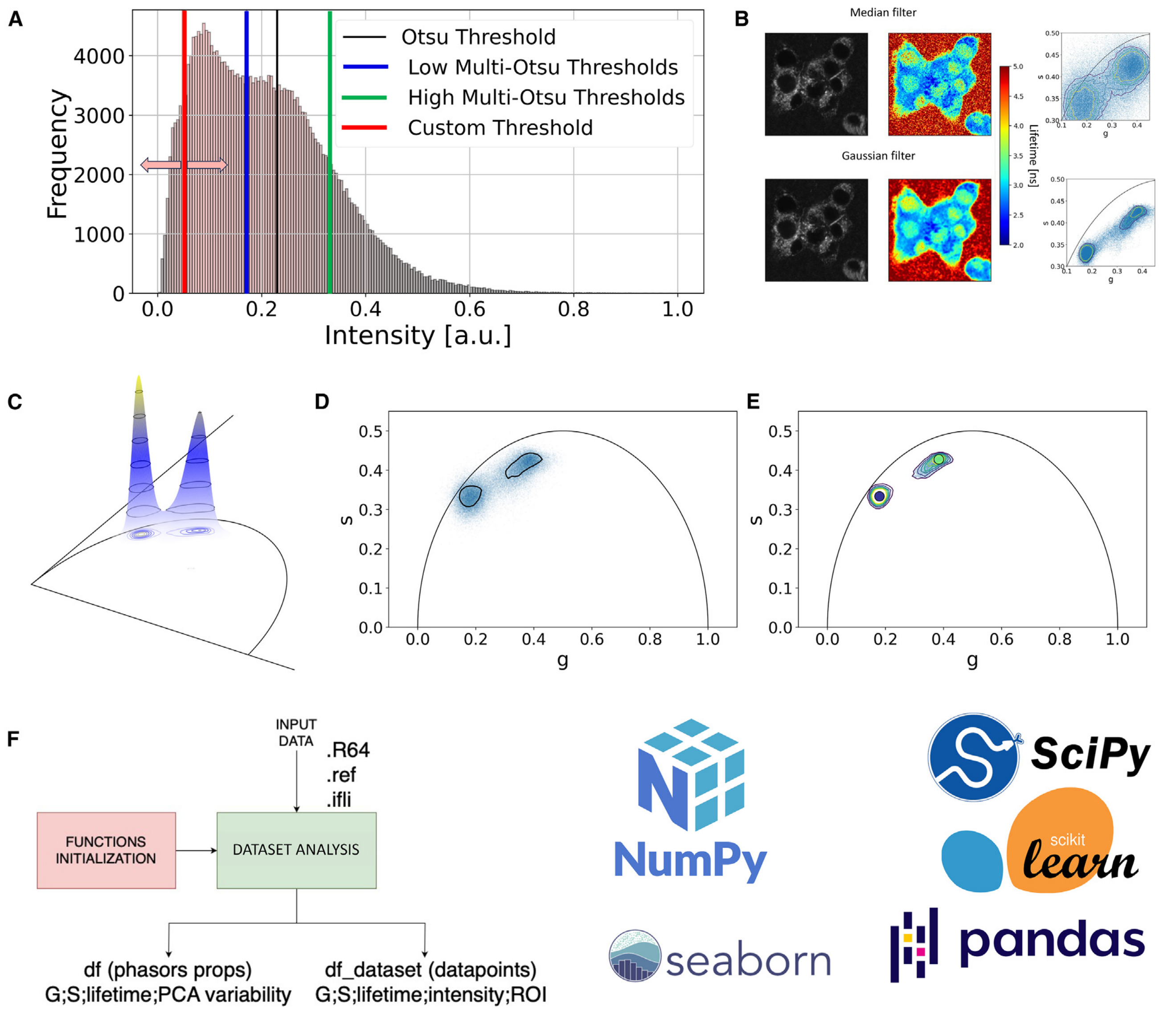

FIGURE 2 Analysis process with filtering and relevant pixel selection. (*A*) Automatic detection of relevant pixels based on the set intensity threshold (custom, Otsu, or multi-Otsu threshold). (*B*) Spatial filtering of pixels using a median or Gaussian filter with user-defined strength (i.e., 3 x 3 median filter; 1σ Gaussian filter). As it can be seen, it is of the main importance to visually check the effect of spatial filtering as it may lead to a loss of resolution. (*C*) 3D visualization of multiphasor-FLIM signal. (*D*) Distinct ROIs identification from phasor-FLIM signal in a single sample measurement. (*E*) Phasors' extraction from phasor-FLIM-identified ROI. (*F*) The primary objective of the code's main block is to preprocess and organize data into user-friendly datasets, facilitating subsequent manipulation in subsequent modules. (The data shown are representative of a cellular phasor of the INS1-E cell line and its extracellular medium upon liposomal irinotecan administration).

Evolution of phasor-FLIM signals

FLIM is frequently employed, in light of its sensitivity to chemical, physical, and biological variations. Fig. 3 *A* shows these effects considering the phasor position of irinotecan at physiological pH as a reference. A basification of the chemical environment results in longer irinotecan characteristic lifetime signal; irinotecan metabolic enzymatic cleavage into the very potent SN-38 compound leads to shorter lifetime, whereas irinotecan liposomal encapsulation (Onivyde liposomal formulation) leads to a combination of physical states (16) and interactions with the liposomal membrane that promote longer lifetime values. Interestingly, both metabolic cleavage and encapsulation lead to multiexponential phasors (hence not lying on the semicircle in the phasor plot) since the molecule is not found in a single physical state. In fact, as shown in Fig. 3 *B*, SN-38 shows an intrinsically multiexponential phasor as the result of a very dynamic chemical equilibrium (52). The code allows the evolution of the FLIM signal for each of the three scenarios mentioned above to be monitored (see Table S1). However, to keep things straightforward, herein we focus on

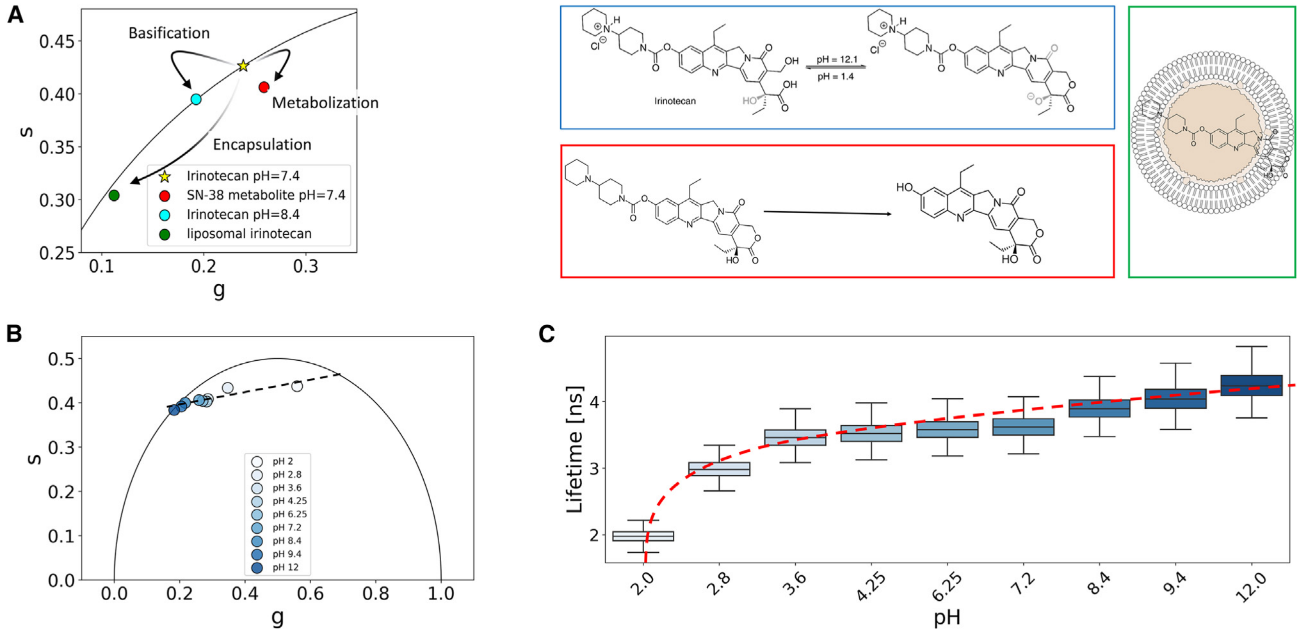

FIGURE 3 Phasor-FLIM detection and analysis of changes in lifetime. (*A*) Phasor-FLIM promptly detects changes in lifetime values stemming from chemical alterations (e.g., basification, *depicted in cyan*), biological transformations (e.g., metabolic cleavage, *highlighted in red*), or physical adaptations (e.g., nanoscale encapsulation, *in green*). In this context, irinotecan (*marked with a star*) at physiological pH serves as a reference point. (*B*) The evolution of SN-38 within the phasor plot is elegantly portrayed, illustrating its response across varying pH levels. (*C*) Depicting the pH against lifetime nonlinear curve of SN-38, the representation reveals the intricate relationship. Utilizing the seaborn library, the code generates a boxplot derived from data frames, meticulously stored within the code.

tracking the lifetime of SN-38 in relation to pH (for SN-38 and irinotecan values, see Fig. S2; Table S2). This choice is made because the change in irinotecan lifetime is less apparent, as outlined in Fig. S2. We initially employed a linear interpolation fit as it appears visually that it is a suitable model based on the data. However, fitting the SN-38 signal in the phasor plot with a linear interpolation ($R^2 \approx 0.80$) is not very satisfactory. It is rather more convenient to move out of the phasor plot in one variable (i.e., pH) against lifetime space to appreciate a proper nonlinear fit (power law $-R^2 \approx 0.98$). As shown in the boxplot of Fig. 3 *C*, the code stores all the data points, relying on pandas data frames, and those can be used to visualize lifetime distributions, in this case as a function of pH. The code stores two different data frames, one containing the data of each data point named df_dataset and one another accounting for the properties of phasors named df. Both can be saved as csv format and accessed outside of Google Colab.

Morphological and statistical analysis: A glimpse into cellular analyses

Once all the ROIs have been detected, the code enables morphological analysis on selected ROIs of each sample, chosen through a drop-down menu. The analysis includes computing lifetime and potentially its average weighted over intensity values by determining the phasor barycenter, along with respective standard deviations. Additionally, it calculates G and S coordinates with corresponding errors, principal component analysis variability ratio, and the number of points sampled to define the phasor. Identifying distinct lifetime regions in a single image is often crucial, so the code facilitates an analysis of intensity, lifetime, and clustering mapping of the sample. Clustering is performed using a Gaussian mixture model, which is recommended for phasor-FLIM data due to the inherent presence of a normal component in phasor distributions stemming from noise in measurements (53). The users have the flexibility to manually specify the number of clusters, with the default set to two clusters.

Application to cell metabolism is reported in Fig. 4 using insulinoma 1E cells (INS-1E). INS-1E cells have been extensively characterized as a mammalian rat cell line that stores and secretes insulin in response to a wide range of glucose concentrations, and they are, therefore, widely used to study the mechanisms of insulin secretion (5,55). Fig. 4 *A* and *B* presents a comparison of INS-1E autofluorescence lifetime signals after incubation with fresh medium (CTRL) and cytokines (CTKs). The phasor plot reveals a significant shift in multicomponent lifetime (measured as τ_m, modulation lifetime), and there are subpopulations

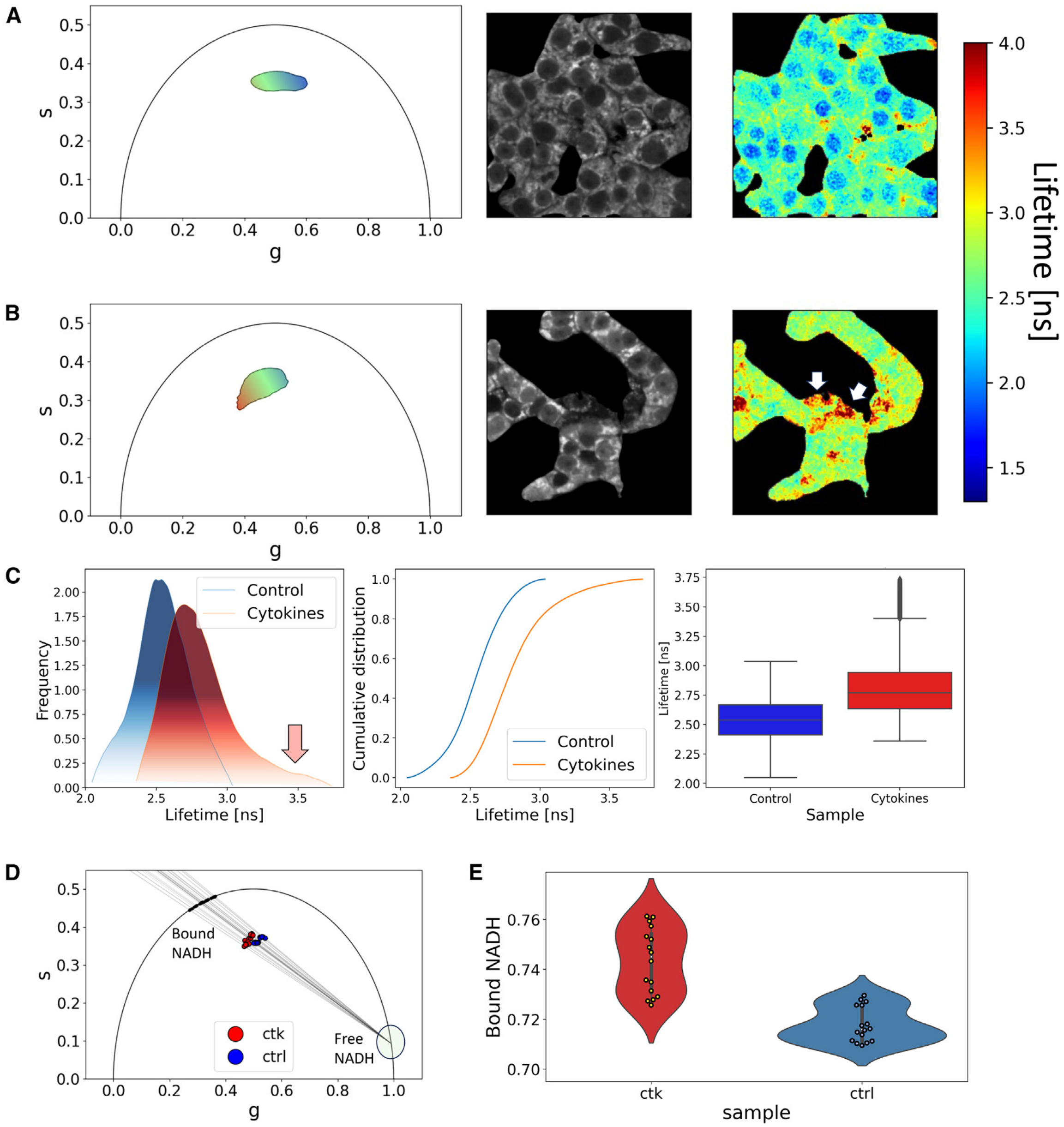

FIGURE 4 Phasor-FLIM comparative statistical and morphological analysis. (*A*) INS-1E autofluorescence lifetime signals after incubation with fresh medium (CTRL) FLIM analysis: in the phasor plot (*left panel*, color-coded), intensity (*middle panel*), and lifetime (*right panel*, color-coded) images. (*B*) INS-1E autofluorescence lifetime signals after incubation with cytokines (CTKs) FLIM analysis: in the phasor plot (*left panel*, color-coded), intensity (*middle panel*), and lifetime (*right panel*, color-coded) images. Arrows indicate the presence of long-lifetime species, previously demonstrated to be the result of reactive oxygen species production. (*C*) Assessment of multimodality using nonparametric fitting (kernel density estimate with Gaussian kernel) on the lifetime data from both CTRL and CTK, with visualization through the cumulative distribution function and boxplot to highlight significant differences (*red arrow* indicates the presence of LLM). (*D*) A linear fit is performed for metabolic assessment by considering the reference free NADH phasor and the cellular phasor. This entails fitting the phasors for both control and cytokine-treated samples. (*E*) The violin plot illustrates the distribution of the fraction of bound NADH in INS1-E cells, revealing significant differences in response to cytokine treatment. (Data are extrapolated from the work of Pugliese et al. (54)).

spreading out of the contour plot core. CTRL and CTK study cases were mapped in lifetime, both on the phasor plot and morphologically, leveraging the data set established by Pugliese et al. (54). This allows us to visually conclude that the CTK treatment has an effect on cellular lifetimes, triggering a shift in both nuclear and cytoplasmic signals toward longer lifetimes. Indeed, as can be viewed in Fig. 4 *B* (and further discussed in (54)), the phasor cluster in the condition of exposure to cytokines clearly shows an elongation toward longer lifetimes with respect to control, presumably owing to the increased contribution of

long-lifetime species, previously demonstrated to be the result of reactive oxygen species production. Specifically, CTK shows a nuclear lifetime subpopulation with a shorter lifetime, whereas CTRL shows a cytoplasmic lifetime subpopulation with a longer lifetime. In Fig. 4 *C*, we assess this multimodality by sampling the computed lifetime (τ_m) distribution using nonparametric fitting (kernel density estimate with Gaussian kernel, bandwidth = 0.01 ns). Subsequently, we conduct two nonparametric statistical tests, Kolmogorov-Smirnoff and Mann Whitney U with Bonferroni correction, to test the null hypothesis that the distributions for CTRL and CTK are the same. The code enables us to perform these statistical tests and reveals significant differences in terms of percentile distributions, central tendency, skewness, and spread (see Table S3). Additionally, Fig. 4 *C* includes the cumulative distribution function and boxplot, which facilitate the visualization of these statistical differences. In conclusion, the morphological and statistical modules demonstrated in Fig. 4 provide a robust framework for conducting FLIM-based assessments, enabling the analysis of cellular environments based on cell autofluorescence signals. In the work of Pugliese et al. (54), we investigated fluorescent molecule metabolism or interactions within the cellular environment. In this particular scenario, the alteration in the FLIM pattern of cellular autofluorescence can be attributed to the increase of both enzyme-bound NAD(P)H molecules and oxidized lipid species (54). To fulfill this objective, Phasor Identifier is equipped with a module for calculating the fraction of NADH that is in the free and bound states (see Fig. S3 for spatial mapping of free and bound NADH levels), consistent with established literature. Fig. 4 *D* illustrates the fundamental concept behind this module: phasors are subjected to linear fitting, originating from the reference phasor representing NADH in its free state, with a lifetime well documented in the literature, typically around 0.37 ns (56,57). This fitting procedure allows the discernment of the NADH bound state's lifetime. In fact, it's important to note that the fluorescence lifetime of bound NADH can vary considerably, ranging from 1 to 9 ns (58,59). This variability is primarily influenced by the specific enzyme to which NADH binds and the presence of allosteric molecules (60). The distribution comparison and analysis are particularly useful for detecting changes in lifetime that may result from interactions or variations in the sample environment, such as temperature fluctuations or pH changes, thereby offering valuable insights into cellular dynamic and metabolic processes. In pursuit of the metabolic shift assessment, Fig. 4 *E* presents a violin plot depicting the proportion of bound NADH in INS1-E cells after cytokine treatment.

From intensity to molar fractions: A case study on liposomal doxorubicin

FLIM can be used to resolve multiexponential signals, where two or more molecular species with distinct lifetimes are coexisting simultaneously. Here we build on a recently demonstrated case study that deals with the presence of three molecular species, i.e., the FDA-approved liposomal nanoformulation Doxoves, which encapsulates doxorubicin as free in solution, crystallized, and associated to the liposome membrane (15). Deconvoluting the multiexponential signal is relatively straightforward, as it involves a linear combination of the three monoexponential phasors characteristic of the pure species (free, crystallized, and bound to membrane). However, it is essential to note that this method provides the "fractional intensity" of each pure physical state: due to differences in molar extinction coefficient (ε) and quantum yield (QY) among pure species, the fractional intensities can be very different from the actual molar fractions. To address this point, we have incorporated a molar fraction module where users can input the name, lifetime (in ns), ε, and QY of each physical state they wish to consider.

In Fig. 5, we present the potential of this approach through a case study involving the storage of Doxoves at different temperatures (4°C and 37°C) for a duration of 120 days. This study provides valuable insights into the behavior of the three molecular species under varying storage conditions (61). In Fig. 5 *A*, in particular, we observe that increasing the temperature to 37°C promotes the transformation of doxorubicin physical state, leading it toward both the membrane-bound and free-in-solution states. The evolution of the phasor plot at 37°C suggests that doxorubicin crystalline structure within Doxoves gets nearly completely dissolved, with Doxoves phasor lying along the trajectory between the membrane-bound and free-in-solution remaining species. This effect becomes evident after 120 days and reflects into a neat change in the average lifetime (Fig. 5 *B*). Indeed, both storage conditions trigger a monoexponential decay in the values of inverse lifetime (1/lifetime), characterized by comparable decay times: $\tau_{4^\circ C} \approx 15$ days and $\tau_{37^\circ C} \approx 19$ days. However, the 4°C storage condition maintains a more consistent lifetime signal in the phasor plot, along with a relatively minor increase in lifetime over the 120-day period (~6%). Conversely, at 37°C under the same storage conditions, a marked increase in lifetime (~36%) is observed. As detailed in Table 2, the fractional intensity of crystallized Doxoves at 37°C experiences a significant decline over 120 days. The Sankey diagram fluxes in Fig. 4 *C* (see also Table 2) reveal changes in the nanoparticle composition, primarily

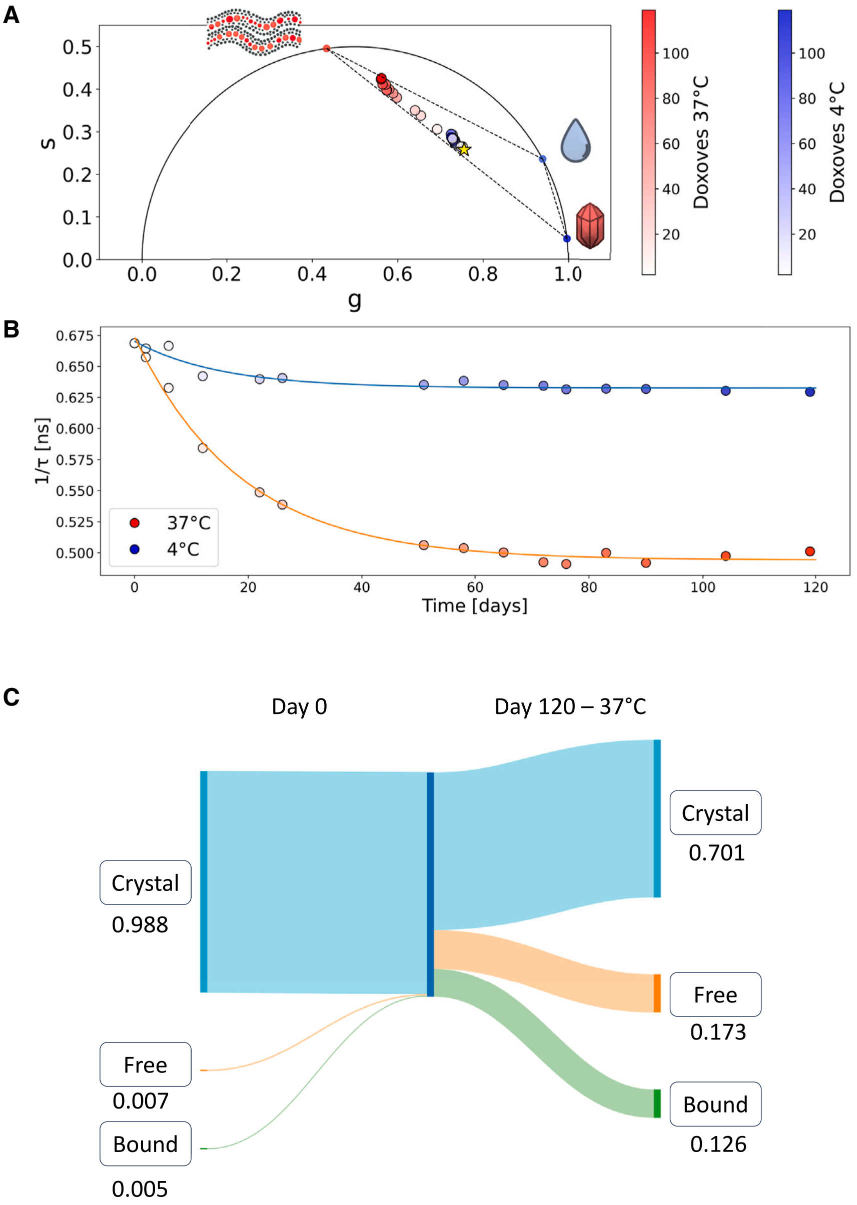

FIGURE 5 FLIM analysis of storage-induced changes in nano encapsulated drugs: liposomal doxorubicin case study. (*A*) The phasor plot showcases the lifetimes of liposomal doxorubicin (Doxoves) within the manufacturer's solution. Monitoring occurred over a 0- to 120-day period under two distinct storage conditions: 4°C (*depicted in the blue palette*) and 37°C (*depicted in the red palette*). The star denotes the initial FLIM signal of Doxoves on day 0. (*B*) The progression of 1/lifetime values over time unveils a consistent monoexponential decay pattern in both storage conditions, despite the significant variations between the two. (*C*) Sankey diagram of the molar compositional changes of Doxoves nanoparticles after 120 days of storage at 37°C.

driven by the dissolution of crystallized doxorubicin. This results in a new molar composition of the liposomal drug (refer to Table S4 for doxorubicin QY and ε values in the relevant physical states).

CONCLUSIONS

In this study, we introduce an innovative approach to capitalize on the computational capabilities of Google Colab to conduct advanced phasor-FLIM analysis. Google Colab grants access to robust computational resources, including GPUs, substantial RAM, and ample disk space, thereby eliminating the need for substantial investments in costly hardware. This is especially beneficial for those seeking a seamless integration of artificial intelligence tools designed for FLIM and working with large datasets. Indeed, within the artificial intelligence-driven FLIM analysis community, significant efforts are underway. These encompass a broad spectrum of advancements, including automatic clustering (36), denoising techniques for fluorescence imaging (62), and enhancements in time domain fitting (41).

Mannam et al. have reviewed the applicability of various machine learning (ML) techniques to fit time domain decay. The majority of them exhibit substantial improvements in processing speed when contrasted with conventional methodologies.

TABLE 2 Fractional intensities and molar fractions of Doxoves in different storage conditions

Sample	Intensity fraction “crystal”	Intensity fraction “free”	Intensity fraction “bound”	Molar fraction “crystal”	Molar fraction “free”	Molar fraction “bound”
Doxoves	0.459	0.125	0.416	0.988	0.007	0.005
Doxoves 4°C (120 days)	0.373	0.163	0.464	0.982	0.011	0.069
Doxoves 37°C (120 days)	0.024	0.227	0.749	0.701	0.173	0.126

All results are obtained from six replicas, and the error on both fractional intensity and molar fraction was systematically $<$0.005 (median filter extent $=$ 3).

In the realm of phasor-FLIM, Vallmjtiana et al. (53) have harnessed ML clustering algorithms, such as the Gaussian mixture model, which are already implemented in the present notebook. Clustering, a subset of ML techniques, finds utility in scenarios where a collection of N-dimensional data points lacks predefined labels. Furthermore, there are notable instances of FLIM-based, label-free NAD(P)H imaging's potential in distinguishing different cell types through the application of artificial neural network-based ML. For their specific biological use case, Sargal et al. focused on the challenge of distinguishing microglia from other glial cell types within the brain (63). Nevertheless, it is important to note that most of the existing software in this domain lacks cloud accessibility, is dependent solely on CPU processing, lacks GPU access crucial for large-scale data processing, and is confined to local hardware limitations. Indeed, our solution empowers researchers to operate within a cloud-based environment, effectively eradicating constraints associated with computational power. Our approach distinguishes itself for two fundamental reasons: it comes without hardware limitations, ensuring flexibility and scalability across a wide spectrum of computing resources; it accommodates both CPU and GPU processing, enabling parallelization. These features closely align with the core objective of our research, which is to trigger a transformative shift in the accessibility and potential of FLIM data. In this context, our demonstration signifies the remarkable flexibility and enhanced accessibility achievable with FLIM data. We further validated by analyzing three compelling case studies involving cellular metabolism, nanoscale drug encapsulation (doxorubicin), and the impact of pH and metabolic cleavage on small fluorescent drugs (irinotecan), showcasing extensive analysis capabilities. The file formats included in this notebook are prevalent within the phasor-FLIM community, spanning both frequency domain and DFD analyses. In the future, we contemplate incorporating the capability to directly convert time domain raw data within the notebook. As of now, such conversions primarily occur within proprietary or freely distributed software platforms.

DATA AND CODE AVAILABILITY

The archived version of the code described in this manuscript can be freely accessed through GitHub (https://github.com/Mariochem92/PhasorIdentifier; https://doi.org/10.5281/zenodo.8282839). The code is extensively documented in the GitHub README file: (https://github.com/Mariochem92/PhasorIdentifier/tree/main#readme), and a test data set including data from this work is accessible at https://zenodo.org/records/10054812.

SUPPORTING MATERIAL

Supporting material can be found online at https://doi.org/10.1016/j.bpr.2023.100135.

AUTHOR CONTRIBUTIONS

M.B. performed research, contributed analytic tools, analyzed data, and wrote the paper; F.C. designed research, provided funds, and wrote the paper.

ACKNOWLEDGMENTS

The authors kindly acknowledge Professor Leonel Malacrida for critical reading of the paper and Dr. Alessandro Rossetta for useful discussions. The authors also acknowledge Dr. Licia Anna Pugliese and Dr. Annalisa Carretta for generously providing the lifetime data analyzed in this study. The Sankey diagram of Fig. 5 was realized in the open environment provided at https://sankeymatic.com/build/. Phasor positions and lifetime values shown in this paper are in keeping and were systematically compared with the outcomes of SimFCS.

The authors acknowledge the support of the European Union by the Next Generation EU project ECS00000017 “Ecosistema dell'Innovazione”Tuscany Health Ecosystem (THE, PNRR, Spoke 4: Nanotechnologies for diagnosis and therapy). The work is supported in part by the European Research Council (ERC) under the European Union's Horizon 2020 research and innovation programme (grant agreement no. 866127, project CAPTUR3D).

DECLARATION OF INTERESTS

The authors declare no conflict of interest.

REFERENCES

1. Scholz, R., R. G. Thurman, …, T. Bücher. 1969. Flavin and pyridine nucleotide oxidation-reduction changes in perfused rat liver. I. Anoxia and subcellular localization of fluorescent flavoproteins. *J. Biol. Chem.* 244:2317–2324.
2. Lakowicz, J. R., H. Szmacinski, …, M. L. Johnson. 1992. Fluorescence lifetime imaging of free and protein-bound NADH. *Proc. Natl. Acad. Sci. USA.* 89:1271–1275.
3. Stringari, C., A. Cinquin, …, E. Gratton. 2011. Phasor approach to fluorescence lifetime microscopy distinguishes different metabolic states of germ cells in a live tissue. *Proc. Natl. Acad. Sci. USA.* 108:13582–13587.
4. Stringari, C., J. L. Nourse, …, E. Gratton. 2012. Phasor Fluorescence Lifetime Microscopy of Free and Protein-Bound NADH Reveals Neural Stem Cell Differentiation Potential. *PLoS One.* 7, e48014.
5. Ferri, G., M. Tesi, …, F. Cardarelli. 2020. Metabolic response of Insulinoma 1E cells to glucose stimulation studied by fluorescence lifetime imaging. *FASEB Bioadv.* 2:409–418.
6. Azzarello, F., L. Pesce, …, F. Cardarelli. 2022. Single-cell imaging of α and β cell metabolic response to glucose in living human Langerhans islets. *Commun. Biol.* 5:1–10.
7. Blacker, T. S., Z. F. Mann, …, M. R. Duchen. 2014. Separating NADH and NADPH fluorescence in live cells and tissues using FLIM. *Nat. Commun.* 5:3936.
8. Okabe, K., N. Inada, …, S. Uchiyama. 2012. Intracellular temperature mapping with a fluorescent polymeric thermometer and fluorescence lifetime imaging microscopy. *Nat. Commun.* 3:705.
9. Uchiyama, S., C. Gota, …, N. Inada. 2017. Intracellular temperature measurements with fluorescent polymeric thermometers. *Chem. Commun.* 53:10976–10992.
10. Suzuki, M., and T. Plakhotnik. 2020. The challenge of intracellular temperature. *Biophys. Rev.* 12:593–600.
11. Murakami, A., K. Nagao, …, M. Umeda. 2022. Cell-autonomous control of intracellular temperature by unsaturation of phospholipid acyl chains. *Cell Rep.* 38, 110487.
12. Puchkov, E. O. 2013. Intracellular viscosity: Methods of measurement and role in metabolism. *Biochem. Moscow. Suppl. Ser. A.* 7:270–279.
13. Liu, T., X. Liu, …, Z. Xu. 2014. Quantitatively Mapping Cellular Viscosity with Detailed Organelle Information via a Designed PET Fluorescent Probe. *Sci. Rep.* 4:5418.
14. Kuimova, M. K., S. W. Botchway, …, P. R. Ogilby. 2009. Imaging intracellular viscosity of a single cell during photoinduced cell death. *Nat. Chem.* 1:69–73.
15. Tentori, P., G. Signore, …, F. Cardarelli. 2022. Fluorescence lifetime microscopy unveils the supramolecular organization of liposomal Doxorubicin. *Nanoscale.* 14:8901–8905.
16. Bernardi, M., S. Giovanni, F. Cardarelli…, 2023. Fluorescence lifetime nanoscopy of liposomal irinotecan Onivyde®: from manufacturing to intracellular processing. *ACS Appl. Bio Mater.* 10:4277–4289.
17. Bernardi, M., and F. Cardarelli. 2023. Nanoscopy on drug-encapsulating nanosystems by phasor-based fluorescence lifetime analysis. *Biophys. J.* 122:278a.
18. Cardarelli, F., F. Beltram, … D. Pozzi. 2022. Determination of the Supramolecular Organization of Encapsulated Molecules by Luminescence Lifetime Analysis.
19. Bernardi, M., M. Vernizzi, …, F. Cardarelli. 2023. Phasor-FLIM-guided unraveling of ATRA supramolecular organization in liposomal nanoformulations. *Nanoscale*.
20. Marcu, L. 2012. Fluorescence Lifetime Techniques in Medical Applications. *Ann. Biomed. Eng.* 40:304–331.
21. Alfonso-Garcia, A., S. Cevallos, …, L. Marcu. 2021. Intraluminal fluorescence lifetime imaging (FLIm) as a diagnostic tool for gastrointestinal disease. *In* Biophotonics Congress 2021 Optica Publishing Group, paper DM3A.4.
22. Maibohm, C., F. Silva, …, J. B. Nieder. 2019. SyncRGB-FLIM: synchronous fluorescence imaging of red, green and blue dyes enabled by ultra-broadband few-cycle laser excitation and fluorescence lifetime detection. *Biomed. Opt Express.* 10:1891–1904.
23. Yasuda, R. 2019. 3 - Principle and Application of Fluorescence Lifetime Imaging for Neuroscience: Monitoring Biochemical Signaling in Single Synapses Using Fluorescence Lifetime Imaging. *In* Neurophotonics and Biomedical Spectroscopy. R. R. Alfano and L. Shi, eds Elsevier, pp. 53–64.
24. Bowman, A. J., C. Huang, …, M. A. Kasevich. 2023. Wide-field fluorescence lifetime imaging of neuron spiking and subthreshold activity in vivo. *Science.* 380:1270–1275.
25. Liput, D. J., T. A. Nguyen, …, S. S. Vogel. 2020. A Guide to Fluorescence Lifetime Microscopy and Förster's Resonance Energy Transfer in Neuroscience. *Curr. Protoc. Neurosci.* 94:e108.
26. Digman, M. A., V. R. Caiolfa, …, E. Gratton. 2008. The Phasor Approach to Fluorescence Lifetime Imaging Analysis. *Biophys. J.* 94:L14–L16.
27. Redford, G. I., and R. M. Clegg. 2005. Polar plot representation for frequency-domain analysis of fluorescence lifetimes. *J. Fluoresc.* 15:805–815.
28. Hanley, Q. S., and A. H. A. Clayton. 2005. AB-plot assisted determination of fluorophore mixtures in a fluorescence lifetime microscope using spectra or quenchers. *J. Microsc.* 218:62–67.
29. Weber, G. 1981. Resolution of the fluorescence lifetimes in a heterogeneous system by phase and modulation measurements. *J. Phys. Chem.* 85:949–953.
30. Jameson, D. M., E. Gratton, and R. D. Hall. 1984. The Measurement and Analysis of Heterogeneous Emissions by Multifrequency Phase and Modulation Fluorometry. *Appl. Spectrosc. Rev.* 20:55–106.
31. Ranjit, S., L. Malacrida, …, E. Gratton. 2018. Fit-free analysis of fluorescence lifetime imaging data using the phasor approach. *Nat. Protoc.* 13:1979–2004.
32. Vallmitjana, A., B. Torrado, …, E. Gratton. 2020. Blind Resolution of Lifetime Components in Individual Pixels of Fluorescence Lifetime Images Using the Phasor Approach. *J. Phys. Chem. B.* 124:10126–10137.
33. Vallmitjana, A., A. Dvornikov, …, E. Gratton. 2020. Resolution of 4 components in the same pixel in FLIM images using the phasor approach. *Methods Appl. Fluoresc.* 8, 035001.
34. Jeong, S., D. A. Greenfield, …, C. L. Evans. 2020. Time-resolved fluorescence microscopy with phasor analysis for visualizing multicomponent topical drug distribution within human skin. *Sci. Rep.* 10:5360.
35. Williams, G. O. S., E. Williams, …, M. Bradley. 2021. Full spectrum fluorescence lifetime imaging with 0.5 nm spectral and 50 ps temporal resolution. *Nat. Commun.* 12:6616.
36. Scipioni, L., A. Rossetta, …, E. Gratton. 2021. Phasor S-FLIM: a new paradigm for fast and robust spectral fluorescence lifetime imaging. *Nat. Methods.* 18:542–550.
37. Malacrida, L. 2023. Phasor plots and the future of spectral and lifetime imaging. *Nat. Methods.* 20:965–967.
38. Gottlieb, D., B. Asadipour, … C. Stringari. 2023. FLUTE: a Python GUI for interactive phasor analysis of FLIM data. 2023.03.31.534529.
39. Schrimpf, W., A. Barth, …, D. C. Lamb. 2018. PAM: A Framework for Integrated Analysis of Imaging, Single-Molecule, and Ensemble Fluorescence Data. *Biophys. J.* 114:1518–1528.
40. Rossetta, A. 2023. The bright future of fluorescence lifetime analysis. *In* Reporters, Markers, Dyes, Nanoparticles, and Molecular Probes for Biomedical Applications XIV SPIE, pp. 19–22.

41. Héliot, L., and A. Leray. 2021. Simple phasor-based deep neural network for fluorescence lifetime imaging microscopy. *Sci. Rep.* 11, 23858.
42. Smith, J. T., R. Yao, …, X. Intes. 2019. Fast fit-free analysis of fluorescence lifetime imaging via deep learning. *Proc. Natl. Acad. Sci. USA.* 116:24019–24030.
43. Gratton, M. A. D., and Enrico. 2014. The phasor approach to uorescence lifetime imaging: Exploiting phasor linear properties. *In* Fluorescence Lifetime Spectroscopy and Imaging CRC Press.
44. Malacrida, L., S. Ranjit, …, E. Gratton. 2021. The Phasor Plot: A Universal Circle to Advance Fluorescence Lifetime Analysis and Interpretation. *Annu. Rev. Biophys.* 50:575–593.
45. Ranjit, S., A. Dvornikov, …, E. Gratton. 2015. Imaging Fibrosis and Separating Collagens using Second Harmonic Generation and Phasor Approach to Fluorescence Lifetime Imaging. *Sci. Rep.* 5, 13378.
46. Blaine, J., L. Lanzano, …, T. Lei. 2011. Dynamic Imaging of the Sodium Phosphate Cotransporters. *Adv. Chron. Kidney Dis.* 18:145–150.
47. Ranjit, S., A. Dvornikov, …, E. Gratton. 2016. Characterizing fibrosis in UUO mice model using multiparametric analysis of phasor distribution from FLIM images. *Biomed. Opt Express.* 7:3519–3530.
48. Pachitariu, M., and C. Stringer. 2022. Cellpose 2.0: how to train your own model. *Nat. Methods.* 19:1634–1641.
49. Stringer, C., T. Wang, …, M. Pachitariu. 2021. Cellpose: a generalist algorithm for cellular segmentation. *Nat. Methods.* 18:100–106.
50. Shirshin, E. A., M. V. Shirmanova, …, M. O. Scully. 2022. Label-free sensing of cells with fluorescence lifetime imaging: The quest for metabolic heterogeneity. *Proc. Natl. Acad. Sci. USA.* 119, e2118241119.
51. Ung, T. P. L., S. Lim, …, C. Stringari. 2021. Simultaneous NAD(P)H and FAD fluorescence lifetime microscopy of long UVA–induced metabolic stress in reconstructed human skin. *Sci. Rep.* 11, 22171.
52. Serrano, L. A., Y. Yang, …, S. Guldin. 2018. pH-Mediated molecular differentiation for fluorimetric quantification of chemotherapeutic drugs in human plasma. *Chem. Commun.* 54:1485–1488.
53. Vallmitjana, A., B. Torrado, and E. Gratton. 2021. Phasor-based image segmentation: machine learning clustering techniques. *Biomed. Opt Express.* 12:3410–3422.
54. Pugliese, L. A., V. De Lorenzi, …, F. Cardarelli. 2023. Unveiling nanoscale optical signatures of cytokine-induced β-cell dysfunction. *Sci. Rep.* 13, 13342.
55. Asfari, M., D. Janjic, …, C. B. Wollheim. 1992. Establishment of 2-mercaptoethanol-dependent differentiated insulin-secreting cell lines. *Endocrinology.* 130:167–178.
56. Kolb, D. A., and G. Weber. 1975. Quantitative demonstration of the reciprocity of ligand effects in the ternary complex of chicken heart lactate dehydrogenase with nicotinamide adenine dinucleotide oxalate. *Biochemistry.* 14:4471–4476.
57. Scott, T. G., R. D. Spencer, …, G. Weber. 1970. Synthetic spectroscopic models related to coenzymes and base pairs. V. Emission properties of NADH. Studies of fluorescence lifetimes and quantum efficiencies of NADH, AcPyADH, [reduced acetylpyridineadenine dinucleotide] and simplified synthetic models. *J. Am. Chem. Soc.* 92:687–695.
58. Ma, N., M. A. Digman, …, E. Gratton. 2016. Measurements of absolute concentrations of NADH in cells using the phasor FLIM method. *Biomed. Opt Express.* 7:2441–2452.
59. Jameson, D. M., V. Thomas, and D. M. Zhou. 1989. Time-resolved fluorescence studies on NADH bound to mitochondrial malate dehydrogenase. *Biochim. Biophys. Acta.* 994:187–190.
60. Ranjit, S., L. Malacrida, …, E. Gratton. 2019. Determination of the metabolic index using the fluorescence lifetime of free and bound NADH in the phasor approach. *J. Biophot.* 12, e201900156.
61. Carretta, A., and F. Cardarelli. 2023. Monitoring drug stability by label-free fluorescence lifetime imaging: a case study on liposomal doxorubicin. *J. Phys, Conf. Ser.* 2579, 012009.
62. Dance, A. 2021. Sharper signals: how machine learning is cleaning up microscopy images. *Nature.* 589:318–319.
63. Sagar, M. A. K., K. P. Cheng, …, K. W. Eliceiri. 2020. Machine Learning Methods for Fluorescence Lifetime Imaging (FLIM) Based Label-Free Detection of Microglia. *Front. Neurosci.* 14:931.

Biophysical Reports, Volume *3*

Supplemental information

Phasor identifier: A cloud-based analysis of phasor-FLIM data on Python notebooks

Mario Bernardi and Francesco Cardarelli

Supplementary Information

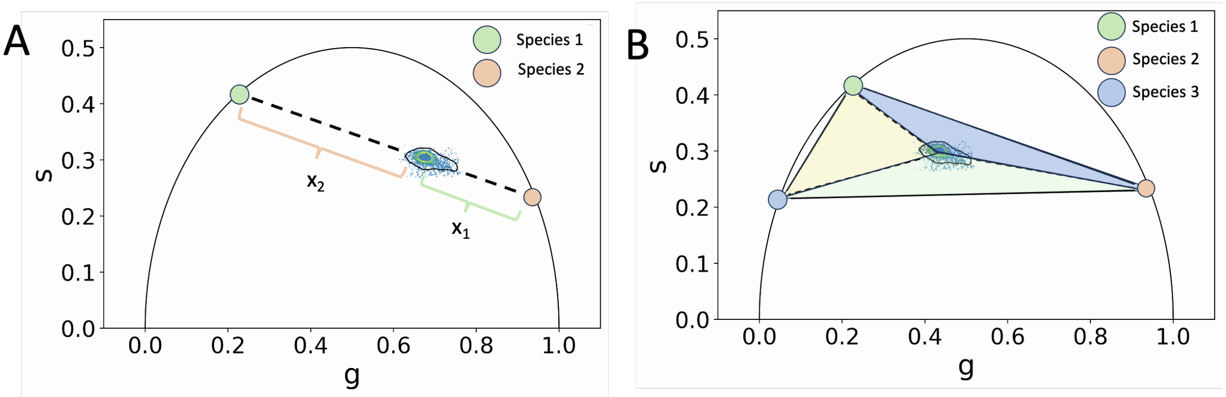

Figure S1. Schematic representation of the graphical approach for assessing intensity fractions of coexisting species knowing the phasor position of pure species a priori. A) In the case of two coexisting species, a linear approach enables straightforward calculation of the intensity fraction by considering the phasor's relative position along the characteristic segment segment. **B)** When dealing with three coexisting species, a linear approach facilitates the calculation of the intensity fraction based on the phasor's relative position within the characteristic triangle.

Sample	G	G std	S	S std	Lifetime [ns]	lifetime std [ns]	sampled points	Contour PCA variability ratio
**irinotecan pH3.6**	0.245019	0.007738	0.423314	0.006534	3.547728	0.010943	182338	0.581715
**irinotecan pH7.2**	0.238661	0.006967	0.421255	0.005676	3.595292	0.009852	170887	0.593733
**irinotecan pH8.4**	0.202396	0.006933	0.394718	0.005882	4.019535	0.009805	154348	0.579785
**SN-38 pH4.25**	0.282524	0.007482	0.403137	0.006235	3.517673	0.010582	244240	0.596726
**SN-38 pH7.2**	0.259005	0.008161	0.406107	0.006879	3.619584	0.011541	296999	0.589467

Table S1. Comprehensive overview of the phasor measurements of Figure 3. The data table shows the diverse impact of physical, chemical, and biological changes on irinotecan, revealing alterations in the FLIM signal.

pH	SN-38 lifetime [ns]	Irinotecan lifetime [ns]
**2.0**	1.98	3.65
**2.8**	2.97	3.61
**3.6**	3.44	3.54
**4.25**	3.50	3.55
**6.25**	3.52	3.56
**7.25**	3.59	3.57
**8.40**	3.89	4.01
**9.40**	4.00	3.99
**12.0**	4.21	4.02

Table S2. Comprehensive overview of the lifetime measurements of SN-38 and Irinotecan in PBS over the wide range of pH values under study, from 2 to 12. The data table shows the diverse impact of pH changes on the lifetime of SN-38 and Irinotecan, revealing alterations in fluorescence behaviour.

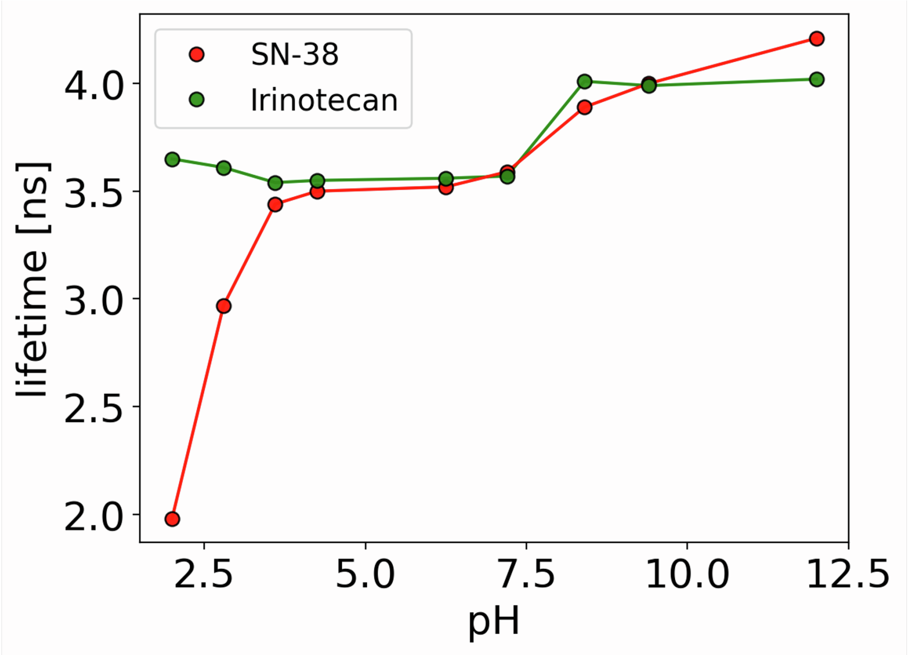

Figure S2. Comparison of the fluorescence lifetime of irinotecan (green) and SN-38 (red) at pH values between 2 and 12. Irinotecan exhibits minimal pH sensitivity compared to its metabolite.

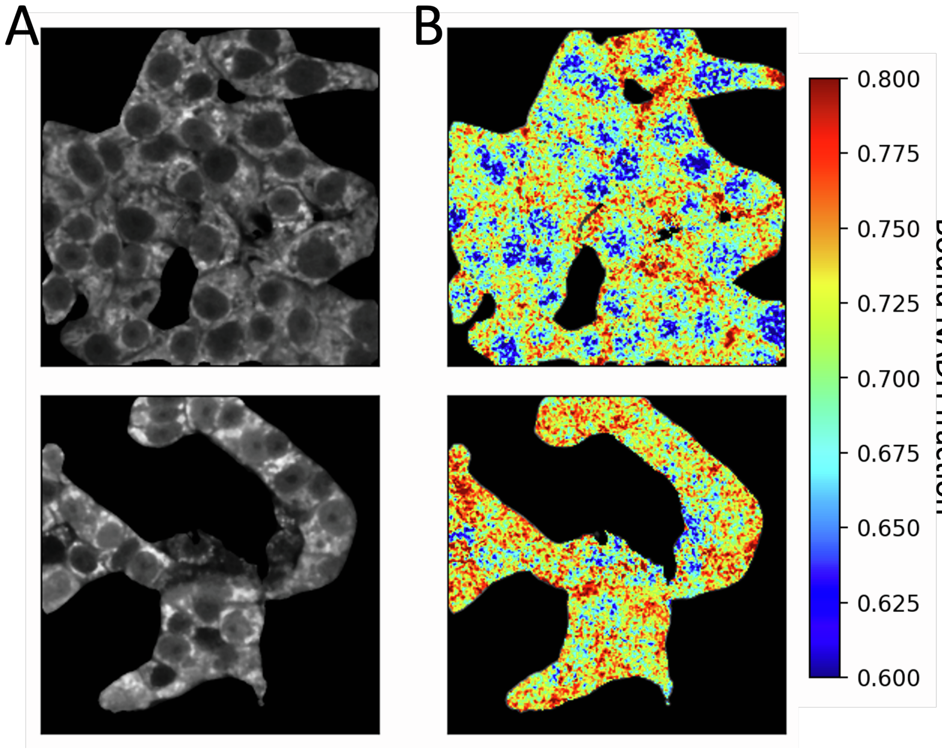

Figure S3: Distribution of Free and Bound NADH within INS1-E Cells Following Cytokine Treatment. A) Two-photon fluorescence intensity images of INS1-E cells under two conditions: control (above) and cytokine-treated (below). **B)** Mapping of the free and bound NADH levels in terms of the fraction of bound NADH in INS1-E cells under two conditions: control (above) and following cytokine treatment (below).

Distribution Parameter	Control	Cytokines
25th Percentile	2.4139	2.6355
50th Percentile	2.5383	2.7703
75th Percentile	2.669	2.9417
Central tendency average	2.5386	2.8161
Spread (Standard Deviation)	0.1921	0.2540
Skewness	-0.0434	0.9799
Mann Withney p-value	0.0	
Kolmogorov Smirnov p-value	1.3746e-86	

Table S3. Lifetime distribution data statistical comparison of INS1E cells (control) and Cytokines. Statistical analysis of the lifetime distributions of Figure 4.

Sample	ε [M^{-1} cm^{-1}]	QY %
Crystal doxorubicin	7510 ± 490	0.150 ± 0.004
Membrane-bound doxorubicin	10 340± 35	19.17 ± 0.4
Free-in-solution doxorubicin	10 340± 35	4.23 ± 0.09

Table S4. Quantum yield and molar extinction coefficient of doxorubicin in distinct physical states: crystal, membrane-bound, and free in solution. These values were sourced from reference (1) and were determined at the specific excitation wavelength of 488 nm.

References

1. Tentori, P., G. Signore, A. Camposeo, A. Carretta, G. Ferri, P. Pingue, S. Luin, D. Pozzi, E. Gratton, F. Beltram, G. Caracciolo, and F. Cardarelli. 2022. Fluorescence lifetime microscopy unveils the supramolecular organization of liposomal Doxorubicin. *Nanoscale*. 14:8901–8905.
